# Electrografting of Phenyl Phosphate Layers onto Glassy Carbon for Tuning Catalytic Activity toward the Hydrogen Evolution Reaction

**DOI:** 10.3390/molecules29040835

**Published:** 2024-02-13

**Authors:** Zaynab Atyf, Quentin Lenne, Jalal Ghilane

**Affiliations:** Université Paris Cité, ITODYS, CNRS, UMR 7086, 15 rue J-A de Baïf, F-75013 Paris, France; zaynab.atyf@etu-u-paris.fr (Z.A.); quentin.lenne@u-paris.fr (Q.L.)

**Keywords:** surface modification, electrochemical grafting, electrocatalysis, hydrogen evolution reaction (HER), electrodeposition

## Abstract

In this study, we explored the surface modification of a glassy carbon electrode through the electrografting of 4-Aminophenyl phosphate, which features heteroatoms and ionic properties. The electrochemical grafting process involves reducing in situ-generated diazonium derivatives. The primary objective of this research was to immobilize organic layers and assess their electrochemical and surface properties. Subsequently, the generated surface serves as a template for the electrochemical growth of Pd and Co nanoparticles on functionalized electrodes. The electrocatalytic performances of these hybrid electrodes in driving the hydrogen evolution reaction were investigated. The obtained results indicate an enhancement in the electrocatalytic activity of the modified electrodes, where lower overpotential and higher stability were observed when the catalyst was electrodeposited onto the attached ionic layer. These findings highlight the synergistic effect between the attached phenyl phosphate moieties and electrocatalysts.

## 1. Introduction

Modifying material properties is a crucial and challenging step in optimizing their performance, functionality, and durability for intended applications. Surface modification is the most straightforward method for achieving this requirement by altering the outermost layer of a material to provide specific functionalities or enhance its interaction with the environment [1]. The most relevant methods for creating thin organic layers are called self-assembled monolayers (SAMs) and electrochemical-assisted approaches [2,3]. While the SAM technique provides a well-defined monolayer structure that profits from the high affinity of specific functionalized groups toward a solid surface, electrochemical approaches generate radicals from an oxidizable or reducible group that can be attached to a conductive substrate [4,5]. Among the various available anchoring groups, diazonium groups are one of the best candidates for generating a thin and compact film, which has been largely used in different applications such as molecular electronics, sensors, and smart surfaces [6,7]. This method electrochemically reduces diazonium derivatives to covalently attach functional groups to a conductor or semiconductor substrate [8]. Electrochemically-assisted grafting offers a wide range of molecules and substrate choices, enabling diverse applications [6]. Electrochemical grafting was also extended to amino–phenyl derivatives using in situ generation of the corresponding diazonium [9,10]. This method involves simple reagents for the diazotization reaction and does not require the isolation and purification of the diazonium salt. The electrochemical grafting of in situ-generated diazonium cations displayed identical properties compared to those using isolated diazonium salt dissolved in acetonitrile or aqueous acid solution [11].

Electrocatalysis holds significant promise for enhancing the efficiency and selectivity of energy conversion and storage devices, including fuel cells, electrolyzers, and batteries [12,13,14]. Additionally, it contributes to the production of sustainable fuels such as hydrogen, playing a pivotal role in the transition to a low-carbon economy [15]. The development of efficient, stable, and cost-effective electrocatalysts remains imperative. In contemporary research, there is a notable emphasis on enhancing catalytic performance, especially the hydrogen evolution reaction for hydrogen production [16,17]. Given the sluggish nature of this reaction, an efficient approach is needed to reduce the reaction overpotential while maintaining stability and durability over time. Previous research has predominantly focused on modulating catalyst properties and investigating the impact of catalyst size, shape, and chemical composition [18,19]. Recently, chemically modified catalysts with thin organic layers were proposed as a promising approach to modulating electrocatalytic activity [20]. However, this approach is at an early stage and more research efforts are needed. Within this context, a novel approach has been proposed, which involves immobilizing thin layers or polymer brushes based on ionic liquid monomers onto glassy carbon followed by the electrochemical growth of the catalyst [21,22]. The modified glassy carbon surface serves as a template for the growth of metallic nanoparticles. As a result, the generated hybrid catalyst displays a higher electrocatalytic performance and stability toward the hydrogen evolution reaction. These performances have been attributed to several effects including the nanostructure of the attached layer, the presence of nitrogen atoms that induce a high electron density and, consequently, favor hydrogen adsorption, and a higher electron transfer between the catalyst and the electrode surface [22].

In this study, electrochemical grafting through cyclic voltammetry was employed to immobilize the phenyl phosphate monosodium layer onto glassy carbon. The generated surface was characterized by surface analysis and probing the electron transfer properties of the redox probe to determine the chemical composition. Then, the generated surface was employed as a platform for the electrochemically-assisted growth of palladium and cobalt nanoparticles. This process led to the generation of hybrid materials containing Pd or Co nano-particles supported by an organic ionic layer. The nanoparticle size distribution was investigated by scanning electron microscopy, and surface analysis was performed to identify ionic layers and NP oxidation states. The quantity of electrochemically deposited NPs was determined by the electrochemical method, which helped estimate the catalyst mass loading. Finally, the electrocatalytic activity of the resulting material was investigated to drive the hydrogen evolution reaction.

## 2. Results and Discussion

### 2.1. Electrografting of 4-Diazoniumphenyl Phosphate onto Glassy Carbon

**Scheme 1:**
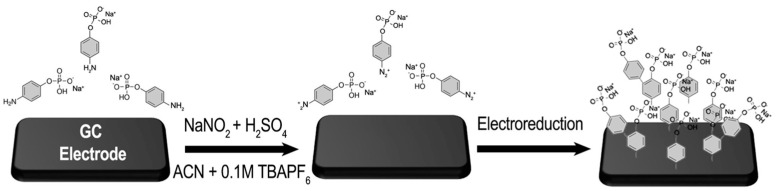
Scheme illustrating the in situ-generated diazonium salt followed by its electrochemical grafting.

The initial phase of the study involved investigating the electrochemical grafting of the corresponding diazonium of 4-Aminophenyl phosphate monosodium salt, which is generated in situ in the electrochemical cell. Firstly, 1 mM of 4-Aminophenyl phosphate monosodium and 0.1 M tetrabutylammonium hexafluorophosphate (TBAPF_6_) were dissolved in acetonitrile. After adding H_2_SO_4_ and sodium nitrite NaNO_2_, a diazotization reaction occurred, leading to the conversion of the amino function into a diazonium one (Scheme 1) [9,10,23].

**Figure 1:**
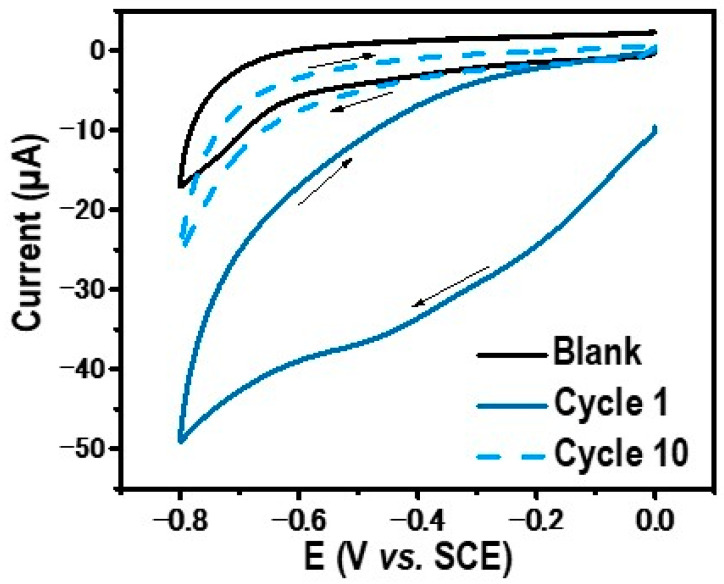
Black line: CV recorded on GC in ACN containing 0.1 M TBAPF_6_ and 1 mM of 4-Aminophenyl phosphate monosodium. Blue and dashed lines: 1st and 10th voltammetric cycle recorded on GC after adding NaNO_2_ and H_2_SO_4_ to generate a diazonium (^+^N_2_–Ph−PO_4_) scan rate of 100 mV·s^−1^.

The electrochemical grafting process was conducted through cyclic voltammetry scanned from 0 to −0.8 V/SCE. The resulting curves are depicted in Figure 1.

**Figure 2:**
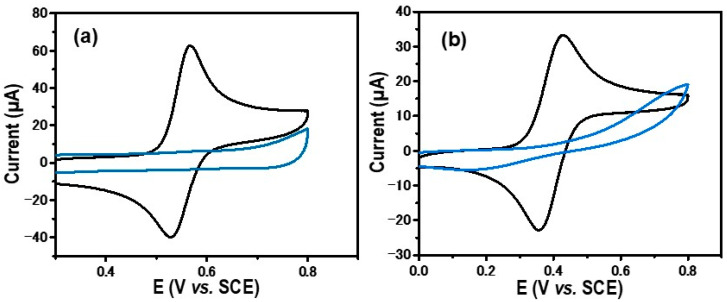
Cyclic voltammetry of bare GC (black) and functionalized electrode GC/Ph−PO_4_ (blue) in (**a**) 0.1 M H_2_SO_4_ aqueous solution containing 1 mM of dopamine and (**b**) 0.1 M TBAPF_6_ acetonitrile solution containing 1 mM of ferrocene (scan rate 100 mV·s^−1^).

In the first cycle, a broad reduction peak located at −0.45 V/SCE was observed and attributed to a reduction in in situ-generated diazonium. With successive cycles, this reduction peak disappears, and no further faradaic current is observed. The decline in the current observed in the tenth cycle suggests that electron transfer from the electrode to the diazonium is completely inhibited by the layers formed in the previous scan. This phenomenon is commonly noted during diazonium reductions on the electrode surface [24]. One must note that before the addition of the diazotization reactants H_2_SO_4_ and NaNO_2_, no electrochemical redox signal was observed. Only a capacitive current was observed between 0 and −0.8 V/SCE (black line in Figure 1). Specifically, diazonium is reduced in the vicinity of the GC electrode to form a radical phenyl derivative, which is highly reactive and subsequently attaches to the electrode surface (Scheme 1). These results strongly indicate the formation of a thin organic film on the electrode surface. To confirm the electrografting of the layer, the modified electrode GC/Ph−PO_4_ underwent electrochemical testing in the presence of ferrocene and dopamine as probes. Before this electrochemical characterization, the modified GC electrodes were rinsed and sonicated in acetonitrile for 3 min to remove weakly adsorbed species. These two probes exhibited distinct electron transfer mechanisms: an outer sphere for ferrocene and an inner sphere for dopamine. Outer-sphere electron transfer can occur within a range of up to 5 nm from the electrode surface through a tunneling effect, while inner-sphere electron transfer occurs after direct contact between the redox probe and the electrode surface, being more sensitive to the surface state [25]. The bare glassy carbon (black line) and modified electrode (blue line) responses in an electrolytic solution containing 1 mM of ferrocene or 1 mM of dopamine are presented in Figure 2a and Figure 2b, respectively.

Regarding the dopamine redox probe, the recorded CV on the bare GC displays a reversible redox system with a potential peak-to-peak separation (ΔEp) close to 30 mV, as was expected for the 2e^−^ transfer redox process under diffusion control (Figure 2a, black line). However, no discernible redox response is observed for dopamine (Figure 2a, blue line) when using the functionalized GC/Ph−PO_4_. The absence of the dopamine redox signal related to the presence of the attached layer, which blocks the electron transfer from the electrode to dopamine. As a redox probe, ferrocene’s electrochemical response in an unmodified GC electrode exhibited a reversible redox signal with ΔEp = 60 mV, confirming that the electron transfer is under diffusion control (Figure 2b, black line). The ferrocene response recorded on the modified electrode (GC/Ph−PO_4_) (Figure 2b, blue line) shows a decrease in current with a peak-to-peak separation of up to 500 mV/SCE, suggesting the presence of a low electron transfer rate. This behavior is attributed to the presence of a thin layer acting as a barrier against the electron transfer of the redox probe [26]. Overall, these findings collectively imply the presence of a thin layer after in situ-generated diazonium was electrochemically reduced.

**Figure 3:**
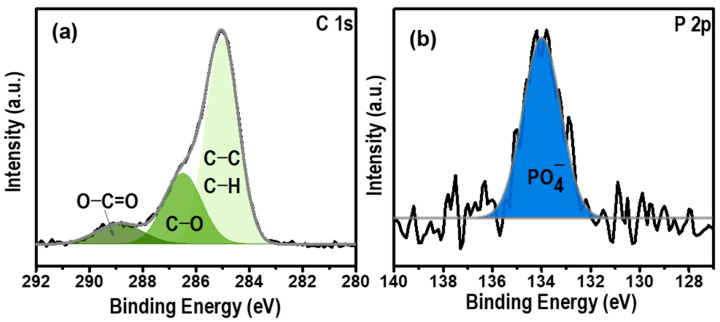
XPS high resolution spectra of modified electrode GC/Ph−PO_4_ for the elements C(1s) (**a**) and P(2p) (**b**).

The chemical composition of the modified surface was analyzed using X-ray photoelectron spectroscopy. The obtained results are illustrated in Figure 3.

The high-resolution C1s spectrum reveals two prominent contributions at 285 eV, attributed to the phenyl group, and 286.5 eV, corresponding to C−O bands from the C bonded to the phosphate group. Additionally, a minor peak at 289 eV was observed and attributed to the presence of the O−C=O band, originating from surface contamination [26]. Moreover, the P 2p_1/2_ spectrum exhibits a peak at 134 eV, characteristic of pentavalent tetracoordinated phosphorus, as in phosphates [27]. Overall, electrochemical and surface investigations collectively affirmed the successful grafting of Ph−PO_4_ molecules onto the GC electrode.

### 2.2. Electrodeposition of Palladium and Cobalt Nanoparticles on GC/Ph-PO_4_

**Figure 4:**
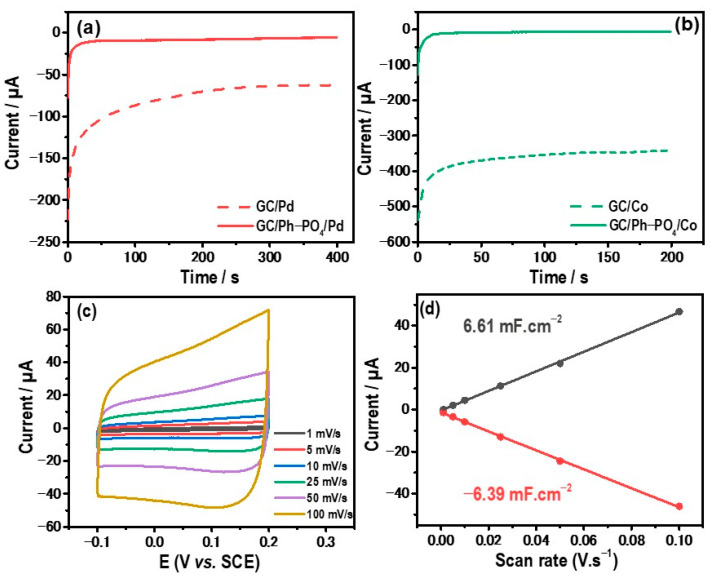
(**a**) Chronoamperometric curves recorded in an aqueous solution containing 0.1 M KCl and 1 mM [Pd(NH_3_)_4_]Cl_2_ on GC (dashed red line) and GC/Ph−PO_4_ (red line). (**b**) Chronoamperometric curves recorded in an aqueous solution containing 0.1 M KNO_3_ and 1 mM Co(NO_3_)_2_ on GC (dashed green line) and GC/Ph−PO_4_ (green line). (**c**) CV in the capacitive region (−0.1–0.2 V/SCE) of GC/Ph−PO_4_/Pd recorded in 0.1 M H_2_SO_4_. (**d**) Variation of the capacitive current as a function of the scan rate (C_dl_ plot).

Following the successful grafting of the ionic layer onto the GC surface, the functionalized electrode served as a platform for the host–guest electrochemical growth of palladium and cobalt. Palladium (Pd) was electrodeposited onto the functionalized layer after immersing the electrode in an aqueous solution containing 0.1 M KCl and 1 mM [Pd(NH_3_)_4_]Cl_2_. Subsequently, a cathodic potential of −0.5 V/SCE was applied for 400 s, resulting in the deposition of Pd onto the modified electrode. For cobalt deposition, the functionalized GC/Ph−PO_4_ was immersed in a 0.1 M KNO_3_ aqueous solution containing 1 mM Co(NO_3_)_2_. A cathodic potential of −1.1V/SCE was then applied for 200 s, allowing the electrochemical deposition of cobalt onto the modified electrode. Figure 4a displays the recorded chronoamperometric (CA) curves of an electrolytic solution containing [Pd(NH_3_)_4_]Cl_2_ on the bare GC (dashed red line) and on the GC/Ph−PO_4_ (red line) electrodes, while Figure 4b shows CA performed in an electrolytic solution containing Co(NO_3_)_2_.

All the CA curves display a similar shape, starting with a rapid current increase due to Pd or Co nuclei formation and growth. The current decays with time and reaches a steady value corresponding to the rise of the diffusion layer thickness and the intersection of diffusion zones [28,29]. The electrodeposition CA curves of Pd and Co display less current on the modified electrode compared to the unmodified GC due to the presence of the Ph−PO_4_ layer, which reduces the growth of Pd and Co. For Pd electrodeposition, the measured charge is −0.45 C/cm^2^ for the GC electrode and −0.05 C/cm^2^ for the modified GC. For Co electrodeposition, the measured charge is −1 C/cm^2^ for the GC and −0.025 C/cm^2^ for the modified electrode. These findings indicate that less charge is passed during the electrochemical deposition of Pd and Co on GC/Ph−PO_4_ compared to unmodified GC. Consequently, fewer amounts of Pd and Co were deposited on the modified GC.

The electrochemical double-layer capacitance (C_dl_) of the electrode GC/Ph-PO_4_/Pd was determined by recording the CV curves at different low scan rates in the capacitive region (Figure 4c). Figure 4d displays the plot of the capacitive current as a function of the scan rate, showing linear variation. The measured C_dl_ for GC/Ph-PO_4_/Pd is around 6 mF.cm^−2^, which is higher than GC/Ph−PO_4_ (0.5 mF.cm^−2^). The change in C_dl_ is linked to the electrode’s active surface area. The electrochemical active surface area (ECSA) was estimated from C_dl_ divided by the specific capacitance (40 µF.cm^−2^). The generated hybrid electrode GC/Ph−PO_4_/Pd displays a ratio C_dl/_C_s_ of about 150, resulting in an active area of 6 cm^2^.

**Figure 5:**
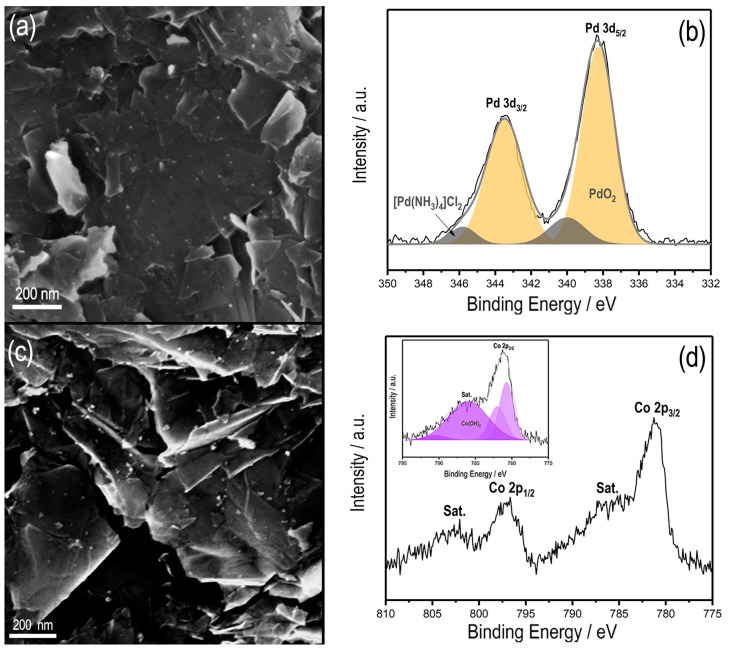
SEM images of (**a**) GC/Ph−PO_4_/Pd, (**c**) GC/Ph−PO_4_/Co, (**b**) XPS survey spectra of the Pd 3d region of GC/Ph−PO_4_/Pd, (**d**) XPS survey spectra of the Co 2p region of GC/Ph−PO_4_/Co.

The hybrid electrodes GC/Ph−PO_4_/Pd and GC/Ph−PO_4_/Co were then characterized by scanning electron microscopy and XPS. For the GC/Ph−PO_4_/Pd SEM images depicted in Figure 5a, bright spots are visible, indicating the presence of Pd nanoparticles. The nanoparticle size distribution was estimated using Image J software, leading to an average nanoparticle diameter of 10 ± 3 nm. Notably, the Pd nanoparticle size is smaller than those deposited onto the bare GC electrode, which had an average diameter of 100 nm [22]. XPS analysis further confirmed the presence of a thin organic layer post−Pd deposition. The peak at 134 eV, attributed to the phosphate group, remained distinctly visible, affirming that the layer remained unaltered during the Pd electrodeposition process.

In the high-resolution spectra of the Pd 3d region (Figure 5b), a predominant peak at 338.3 eV (Pd 3d_5/2_) appears and corresponds to PdO_2_, along with its associated peak Pd 3d_3/2_ at 344 eV [30]. Both SEM and XPS investigations collectively corroborated the successful grafting of the organic layer, followed by the electrodeposition of Pd nanoparticles. These conditions produced a hybrid surface characterized by lower mass loading, smaller nanoparticles, and the presence of an oxidized Pd species, namely PdO_2_.

In the SEM images of GC/Ph-PO_4_/Co (Figure 5c), spherical nanoparticles are evident with an average size of 13 nm, which is smaller than those deposited onto the bare GC electrode (with an average size of about 100 nm). The XPS spectrum of the Co 2p region (Figure 5d) reveals peaks at 781 eV and 797 eV attributed to Co 2p_3/2_ and Co 2p_1/2_, respectively. In addition, peaks with bond energies of 786.2 eV and 802 eV are the satellite peaks of Co 2p_3/2_ and Co 2p_1/2_, respectively [31]. The deconvoluted Co 2p_3/2_ spectrum (inset Figure 5d) displays two contributions located at 780.7 and 782.2 eV, which is consistent with the presence of Co(OH)_2_ [32,33]. Additional proof of the Co^2+^ oxidation state is the presence of characteristic satellite peaks at 786.2 and 802 eV [34]. These results confirm the covalently grafted Ph−PO_4_ layer onto the GC surface and the electrodeposition of Pd and Co in their oxide forms PdO_2_ and Co(OH)_2_.

### 2.3. Electrochemical Activity

The generated hybrid catalysts GC/Ph−PO_4_/Pd and GC/Ph−PO_4_/Co were examined for the hydrogen evolution reaction in a 0.1 M H_2_SO_4_ aqueous solution using linear sweep voltammetry (LSV) at a scan rate of 10 mV/s. Electrocatalytic activity of metal catalysts, Pd and Co, deposited onto GC/Ph−PO_4_ was compared to Pd and Co deposited on bare GC electrodes. It is worth mentioning that no binder was used in any of the electrocatalytic experiments, such as Nafion.

**Figure 6:**
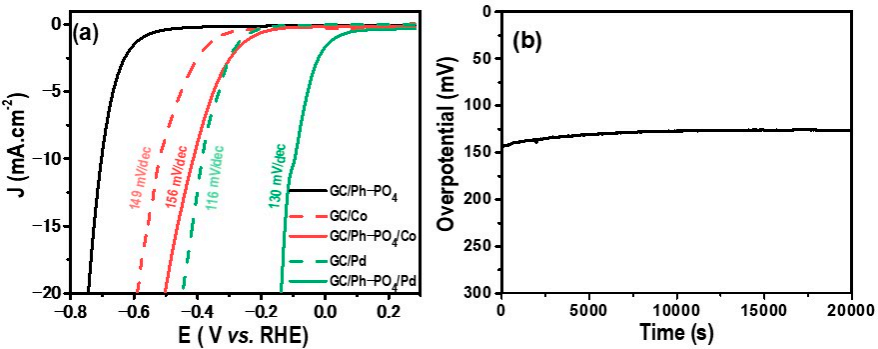
(**a**) HER polarization curves in a 0.1 M H_2_SO_4_ aqueous solution at 10 mV/s for GC/Pd (dashed green line), GC/Ph−PO_4_/Pd (full green line), GC/Co (dashed red line), GC/Ph−PO_4_/Co (full red line), and GC/Ph−PO_4_ (black line). (**b**) Plot of overpotential versus time for the GC/Ph−PO_4_/Pd catalyst at a constant cathodic current density of −10 mA cm^−2^ in 0.1 M H_2_SO_4_ aqueous solution.

The polarization curve depicted in Figure 6a during the LSV exhibits characteristic H^+^ reduction behavior, evident from the sharp rise in reduction current.

The electrocatalytic activity of GC/Co and GC/Ph−PO_4_/Co was investigated, as shown in Figure 6a. Hybrid catalysts exhibit superior activity compared to cobalt deposited onto a bare GC electrode. Specifically, the onset potential of GC/Co (potential was −387 mV at a current density of −2 mA·cm^−2^), with an overpotential of approximately 518 mV to reach −10 mA·cm^−2^. By contrast, GC/Ph−PO_4_/Co displayed an onset potential of −281 mV and an overpotential of 413 mV to reach −10 mA·cm^−2^. These results indicate that the presence of an organic layer enhances the catalysts’ overall activity.

The catalytic performance of the hydrogen evolution reaction (HER) is considerably distinct between the GC/Pd and GC/Ph−PO_4_/Pd electrodes. Specifically, the electrodeposited Pd on the bare GC electrode demonstrated an onset potential for HER at −291 mV and an overpotential of 386 mV at −10 mA·cm^−2^. By contrast, GC/Ph−PO_4_/Pd displayed superior catalytic activity, with an onset potential of −10 mV and an overpotential of 96 mV to reach −10 mA·cm^−2^. This considerable overpotential variation emphasizes the positive impact of the organic layer on catalytic performance, resulting in significantly lower overpotentials compared to deposited Pd nanoparticles on bare GC electrodes. The electrochemically attached layer displayed poor electrocatalytic HER activity (black line in Figure 6), confirming that the attached layer containing the “P” heteroatom and ionic properties was insufficient to efficiently drive HER. This result confirms the synergy between the Pd catalyst and the attached layer. Despite the enhanced electrocatalytic activity observed in GC/Ph−PO_4_/Pd compared to GC/Pd, the kinetics of the hydrogen evolution reaction (HER) present a nuanced perspective. HER occurs on the surface of the cathode via a multi-step electrochemical process [35]. Under acidic conditions, the HER process occurs following these reactions:

$$H^{+}+e^{-}+*\to \;H*\;(\text{Volmer step: 120 mV}\cdot {\mathrm{dec}}^{-1})$$

$$H*+\;H^{+}+e^{-}\to \;H_{2}+*\;(\text{Heyrovsky step: 40 mV}\cdot {\mathrm{dec}}^{-1})$$

$$2H*\;\to H_{2}+2*\;(\text{Tafel step: 30 mV}\cdot {\mathrm{dec}}^{-1})$$

where, * corresponds to the electrocatalyst surface site.

No significant difference was observed in the reaction kinetics and mechanism, as shown by the similar Tafel slope value. Tafel plots indicate that GC/Pd displayed a reaction rate of 116 mV/dec, comparable to GC/Ph−PO_4_/Pd (130 mV/dec), which showed that the HER of the catalyst occurred via a Volmer–Heyrovsky mechanism and that the HER is controlled by the first electron transfer step [18,36]. The attached layer did not change HER kinetics but improved its overpotential. Since the attached layer contained phosphides, the negatively charged PO_4_ moieties improved and accepted protons, whereas the Pd catalyst acted as an electron collector. In addition, the presence of Pd deposited on PO_4_ groups decreased the energy barrier for H adsorption and modified the electronic structure [37]. Similar behavior was also observed for GC/Co and GC/Ph−PO_4_/Co, with Tafel slope values of 149 and 156 mV/dec, respectively.

Several factors contributed to the enhanced catalytic activity observed in GC/Ph−PO_4_/Pd [18]. Firstly, the size and shape of the deposited Pd nanoparticles played a crucial role. Notably, smaller nanoparticles were obtained when deposited onto the organic layer compared to those onto the bare GC electrode, leading to an increased electrochemical active surface and improved electrocatalytic activity. Furthermore, the chemical composition of the organic layer, which contained phosphorus, introduced heteroatoms that influenced the catalyst’s performance [38,39].

Based on the SEM images, this process generated hybrid materials characterized by smaller nanoparticle sizes in the ionic layer compared to bare GC. The catalyst amount, or mass loading, is a crucial parameter for catalytic activity. The quantity of deposited Pd and Co can be estimated by integrating chronoamperometric (CA) curves performed during electrochemical deposition. The measured average mass loading for each material stood at five different electrodes. The average mass loading values of Pd and Co electrodeposited onto the GC electrode were 250 and 280 µg.cm^−2^, respectively. However, the average mass loading values of electrodeposited Pd and Co over GC/Ph−PO_4_ were estimated between 20 and 10 µg.cm^−2^, respectively. Comparing different values highlights that the attached ionic layer GC/Ph−PO_4_ considerably lowers the amount of deposited Pd or Co compared to an unmodified GC electrode. Our experimental conditions resulted in mass loading values of the same order for Pd and Co deposited on GC and one order of magnitude lower than those measured by the modified GC electrode (GC/Ph−PO_4_).

The stability of functionalized GC/Ph−PO_4_/Pd was assessed by chronopotentiometry at −10 mA.cm^−2^ for 10 h in acidic media. As illustrated in Figure 6b, the hybrid catalyst exhibited good stability during the test period. Specifically, the overpotential remained stable at 140 mV for 20,000 s.

These results clearly demonstrate that the organic layer influences catalytic activity via its impact on metal deposition and properties or its inherent contribution to the catalytic system. Moreover, these findings indicate that this novel approach is universal and applicable to various metals, such as Pd and Co. It is worth emphasizing that achieving high performance on a functionalized electrode requires low metal mass loading as opposed to the large mass loading observed on a bare GC electrode.

## 3. Materials and Methods

### 3.1. Materials

#### 3.1.1. Chemicals

4-Aminophenyl phosphate monosodium salt was furnished by Sigma–Aldrich. For the metal electrochemical deposition, [Pd(NH_3_)_4_]Cl_2_ and Co(NO_3_)_2_ (supplied by Sigma–Aldrich, Darmstadt, Germany) were used as received. Ferrocene and dopamine (supplied by Sigma–Aldrich) were used as redox mediators. Sulfuric acid (H_2_SO_4_, 18 M) was purchased from Acros Organics (Illkirch, France) and sodium nitrite (NaNO_2_) from Fluka (Darmstadt, Germany). Tetrabutylammonium hexafluorophosphate (TBAPF_6_) from Sigma–Aldrich was used as a supporting electrolyte at 0.1 M in acetonitrile (ACN). All the chemicals used were purchased at the highest available purity.

#### 3.1.2. Surface Characterization

The size distribution and morphology of the nanoparticles were estimated by scanning electron microscopy (SEM) using a Zeiss Gemini (Rueil Malmaison, France) SEM 360 with an acceleration voltage of 7 kV. Size distribution was estimated using Image J software, version 1.52a.

X-ray photoelectron spectroscopy (XPS) measurements were performed using a K-Alpha+ system (ThermoFisher Scientific, East-Grinsted, UK) fitted with a micro-focused and monochromatic Al Kα X-ray source (hν = 1486.6 eV, spot size of 400 μm, 12 kV). The pass energy was set to 150 and 40 eV for the survey and narrow high-resolution regions, respectively. The recorded XPS spectra were calibrated and fitted using Avantage software, version 5.977. Binding energies were cross-referenced with the C1s peak at 285 eV.

#### 3.1.3. Electrochemical Measurements

For the electrochemical experiments, a conventional three-electrode cell was used. A graphite rod (purchased from Alfa Aesar, Schiltigheim, France) was used as an auxiliary electrode. A saturated calomel electrode (SCE) was used as the reference electrode. Glassy carbon (GC) disk electrodes were supplied from IJ Cambria (Llanelli, UK), with a surface area of 0.7 cm^2^, and used as the working electrode.

Electrochemical measurements were performed on a CHI660C potentiostat (CH Instruments, Austin, TX, USA). All the solutions were systematically deoxygenated by bubbling argon before and during the experiments. CVs are presented using the European convention (cathodic potential is negative; cathodic current is negative).

### 3.2. Methods

#### 3.2.1. GC Surface Functionalization

Electrografting on GC electrodes commenced with initial preparation steps. Prior to use, the GC electrodes were polished successively using SiC-paper 5 µm (Struers, Champigny sur Marne, France) and DP-Nap paper 1 µm (Struers, Champigny sur Marne, France) with Al_2_O_3_ 0.3 µm slurry (Struers, Champigny sur Marne, France). After polishing, the electrode was thoroughly rinsed with ultrapure water (18.2 MΩ cm) and subjected to sonication in analytical grade ethanol and acetonitrile for 5 min, respectively.

Prior to GC modification, 1 mM of 4-Aminophenyl phosphate monosodium salt and 0.1 M of TBAPF_6_ were added as supporting electrolytes and dissolved in acetonitrile. Diazonium was generated in the electrochemical cell by adding the necessary reagents for the diazotization reaction. Thus, under an argon flow, 50 µL of aqueous NaNO_2_ (1 M) was added, followed by 80 µL of H_2_SO_4_ (18 M).

After the modification, the electrodes underwent thorough rinsing with acetonitrile and Milli-Q water, respectively. Finally, the modified electrodes were sonicated in acetonitrile for 3 min to eliminate weakly adsorbed species.

#### 3.2.2. Metals Electrodeposition on Functionalized GC Electrodes

Palladium was deposited from a solution containing 1 mM of [Pd(NH_3_)_4_]Cl_2_ and 0.1 M KCl as supporting electrolytes using chronoamperometry at −0.5 V/SCE for 400 s. Cobalt (Co) deposition entailed electrodeposition from an aqueous solution containing 1 mM Co(NO_3_)_2_ in 0.1 M KNO_3_, with chronoamperometry at −1.1 V/SCE for 200 s. The deposited metals underwent characterization with scanning electron microscopy (SEM) and X-ray photoelectron spectroscopy (XPS). Metal mass loading was determined using the following formula:

(1) $$m_{\mu g\cdot {cm}^{-2}\;=\;\frac{C\cdot M}{C_{e-}\cdot n_{e-}\cdot N_{A}\cdot A}}$$

where,

*C* is the charge (*C*) integrated from the chronoamperometric curve;
*M* is the molar mass of the metal;
*C*_*e*−_ is the charge of a single electron (1.6 × 10^−19^ *C*);
*n*_*e*−_ is the number of electrons involved in the reduction (i.e., 2);
*N_A_* is the Avogadro number (6.022 × 10^23^ mol^−1^);
*A* is the surface of the electrode (0.0707 cm^2^).

#### 3.2.3. Electrochemical Activity Investigations

To assess the electrocatalytic activity of the hydrogen evolution reaction, experiments were conducted in a 0.1 M H_2_SO_4_ aqueous solution using linear sweep voltammetry. During the experiments, all electrolytes were maintained in an inert atmosphere. Linear sweep voltammetry (LSV) with a scan rate of 10 mV/s was used for the hydrogen evolution reaction test. The LSV curves were corrected through an ohmic potential drop using the IR compensation test. Tafel plots were subsequently calculated to monitor the kinetic reaction. For HER experiments, the potentials were converted versus the hydrogen reversible electrode (RHE) using the following formula:

E (RHE) = E (SCE) + 0.242 + 0.059 pH(2)

## 4. Conclusions

In summary, this study details the successful functionalization of a glassy carbon electrode through electrochemical grafting using in situ-generated diazonium from 4-Aminophenyl phosphate monosodium. Electrochemical and surface analyses confirmed the grafting of Ph-PO_4_ onto the GC electrode. Subsequently, the thin organic layer served as a platform for the electrodeposition of Pd and Co nanoparticles. The resulting hybrid materials were used for the electrocatalysis of the hydrogen evolution reaction (HER) and exhibited improved catalytic performance. Notably, GC/Ph-PO_4_/Pd demonstrated superior performance and stability compared to GC/Pd despite a lower mass loading of deposited Pd than on the bare GC electrode. The Co hybrid catalyst further validates the versatility of this approach for various metals. This work underlines the potential of surface functionalization based on organic ionic layers containing heteroatoms as a promising strategy for developing high-performance and stable hybrid catalysts.

## Data Availability

No new data were created.
